# HapAsmbl: A reference‐aided pipeline for assembling haplotypes in Nanopore amplicon sequence data of polymorphic populations

**DOI:** 10.1002/aps3.70062

**Published:** 2026-06-12

**Authors:** Ayodele Oluwaseyi Fakoya, Augustine Chen, Rowan Paul Herridge, Richard Colin Macknight, Lynette Ruth Brownfield

**Affiliations:** ^1^ Department of Biochemistry University of Otago Dunedin New Zealand

**Keywords:** *CO* gene, *FT3* gene, haplotype assembly, haplotype diversity, heterozygous perennial ryegrass, multi‐locus long‐read amplicon sequencing, *VRN1* gene

## Abstract

**Premise:**

Advances in long‐read sequencing offer new possibilities to investigate haplotype diversity across multiple genes in plants and other taxa through multi‐locus, long‐read amplicon sequencing (multi‐locus LRAS). Despite this progress, there is a notable absence of dedicated bioinformatics pipelines for assembling diploid haplotypes of heterozygous individuals from such multi‐locus LRAS datasets, which is required for highly polymorphic populations.

**Methods:**

We first evaluated various de novo and reference‐based assembly methods, culminating in a custom pipeline (HapAsmbl) to assemble haplotypes from Oxford Nanopore Technologies (ONT) LRAS data of five flowering genes (*FT3*, *FTL9*, *VRN1*, *VRN2A*, and *VRN2B*) generated from perennial ryegrass, a highly heterozygous species. After verifying the efficacy using a simulated heterozygous dataset, the HapAsmbl pipeline was used to explore haplotype diversity of *CO*, *FT3*, and *VRN1* across multiple ryegrass populations.

**Results:**

HapAsmbl outperformed existing tools by reliably reconstructing diploid haplotypes across multiple loci, enabling efficient haplotype characterization and novel allele discovery in genetically diverse populations.

**Discussion:**

HapAsmbl simplifies haplotype resolution from complex LRAS datasets from heterozygous individuals, allowing routine use of ONT long‐read sequencing for scalable haplotype analysis. HapAsmbl will enable researchers to uncover novel alleles and relate these to phenotype, supporting plant‐breeding efforts in non‐model crops.

Amplicon sequencing is widely used to generate sequence information from specific genomic regions in multiple individuals from one or more populations (Murray et al., 2015). It generally starts with amplifying genomic regions using locus‐specific primers via PCR. The generated amplicons are then sequenced directly or cloned into a plasmid vector, with subsequent sequencing of specific clones (Rafalski, 2002; Ostezan et al., 2021).

In plant research, the selection of an amplicon sequencing approach is primarily influenced by the reproductive behavior of the species being studied (Cogan et al., 2006). For species that are self‐compatible and for which inbreeding over multiple generations has led to high levels of homozygosity, direct amplicon sequencing can be applied. Amplicon sequencing in plants with high levels of heterozygosity, such as self‐incompatible species, is more complex as the sequences of the two alleles within an individual need to be distinguished within the pool of amplicons. Traditionally, this has led to the extra step of cloning amplicons prior to sequencing (Cogan et al., 2006), increasing time and resources and limiting throughput.

The development of high‐throughput DNA sequencing technologies capable of simultaneously sequencing multiple samples in parallel has enhanced the efficiency and scalability of direct amplicon sequencing. Next‐generation short‐read sequencing (NGS) technologies provide high accuracy but can result in uneven coverage of target amplicons (Sato et al., 2019; Gunasekera et al., 2021), complicating the resolution of complex haplotypes. Haplotype assembly using short‐read amplicon sequencing (SRAS) data is generally achieved through two approaches. The first involves phasing single‐nucleotide polymorphisms (SNPs) within a specified region into haplotypes using programs like CandiHap (Li et al., 2023) and geneHapR (Zhang et al., 2023a). A notable drawback of such SNP‐based haplotyping programs is their tendency to produce an excessive number of haplotypes for genes with high levels of variation and their inability to incorporate short insertion‐deletions (indels) or structural variants when inferring haplotypes. In the second approach, haplotypes are directly reconstructed using either a short‐read de novo assembler like CAP3 (Huang and Madan, 1999) or a haplotype‐aware assembler like dipSPAdes (Safonova et al., 2015). However, insufficient overlap among reads from different regions of the target amplicon in SRAS datasets often leads to the assembly of non‐contiguous sequences (Veeckman et al., 2019).

Direct amplicon sequencing with long‐read sequencing technologies developed by Oxford Nanopore Technologies (ONT) and Pacific Biosciences (PacBio) overcome several limitations associated with the sequencing of clones and SRAS to identify diploid haplotypes from heterozygous plants. Long‐read amplicon sequencing (LRAS) enables direct end‐to‐end sequencing of amplicons, facilitating assembly of full‐length haplotypes from both homozygous and heterozygous individuals. However, LRAS is a relatively new and rapidly evolving technique and, therefore, still poses several challenges. First, base error rates of data produced from long‐read technologies, although improving, are comparably higher than those of NGS technologies such as Illumina short‐read sequencing (Amarasinghe et al., 2020). Second, while publicly available repositories such as https://nf-co.re/pipelines and https://labs.epi2me.io/wfindex utilize various containerization tools including conda to provide workflows for assembling various ONT datasets, none of these currently include a dedicated workflow for haplotype‐aware assembly of LRAS datasets. This is further compounded by a lack of heterozygous LRAS benchmark datasets that have been validated by alternative sequencing technologies. This necessitates the creation of artificial heterozygous data from confirmed haploid ONT amplicon datasets, such as mitochondrial *cytochrome oxidase I* (*COI*) sequences from ONT and Sanger sequencing (Maestri et al., 2019).

The goal of this study was to assess the utility of direct multi‐locus LRAS and haplotype‐aware bioinformatics pipelines for assembling and identifying sequence haplotypes in highly heterozygous plant populations. To achieve this, we used perennial ryegrass (*Lolium perenne* L.), an important forage species used to pasture livestock in temperate agricultural regions (Charlton and Stewart, 1999). Perennial ryegrass is self‐incompatible and has a high degree of genetic variation within and between populations (Cresswell et al., 2001; Skøt et al., 2005). Previous analyses into haplotype diversity at ryegrass candidate genes implicated in various agronomic traits, including disease resistance (Xing et al., 2007), nutritive quality (Ponting et al., 2007), shoot morphology (Brazauskas et al., 2010), drought tolerance (Cogan et al., 2006), water‐soluble carbohydrate content (Skøt et al., 2007), and flowering time (Asp et al., 2011; Fiil et al., 2011; Skøt et al., 2011), have predominantly utilized clone‐specific amplicon sequencing, limiting throughput and implementation for large population sizes.

Here, we report on using multi‐locus ONT‐LRAS to generate amplicons for perennial ryegrass flowering‐related genes and the development of a custom pipeline, HapAsmbl, to assemble diploid haplotypes. We first test our pipeline on simulated heterozygous samples of *COI* alleles (Maestri et al., 2019) and then demonstrate the utility and scalability of the multi‐locus ONT‐LRAS and HapAsmbl workflow by surveying the diversity of three genes (*CO*, *FT3*, and *VRN1*) across a large sample size and comparing the haplotypes generated with those obtained from other sources.

## METHODS

### Plant materials

Diploid populations of perennial ryegrass, including the cultivar One^50^ (PGG Wrightson, Christchurch, New Zealand) and unreleased F_2_ populations generated from crosses between various cultivars and geographically diverse germplasm, were used to represent a wide range of allelic variation (Table S1 in Appendix S1, see Supporting Information with this article). Five plants and a pooled leaf sample from One^50^ and from F_2_ family A24983 were used for protocol design, and 14 One^50^ and 141 F_2_ plants from eight populations were used for the haplotype diversity screen (Table S1 in Appendix S1). Plants were grown outdoors in 20‐cm pots at the University of Otago, Dunedin, New Zealand.

### Generation of amplicons

#### Target genes and primer design

Five genes related to flowering time (*FT3*, *FTL9*, *VRN1*, *VRN2A*, and *VRN2B*) were selected for protocol development, while *CO*, *FT3*, and *VRN1* were used for haplotype diversity analysis (gene identifiers are provided in Table S2 in Appendix S1). Primers were designed to amplify near full‐length sequences, except for *VRN1* and *VRN2A* where primers flanked the promoter and 5′ end of the coding region or intron 1 (Figure S1 in Appendix S1). Primer sequences and pairs used in each experiment are provided in Table S3 (Appendix S1).

#### Amplicon generation

Genomic DNA (gDNA) was extracted from 50–100 mg of leaf tissue of individual plants or pooled leaf samples following Edward's protocol (Edwards et al., 1991). PCR reactions used 100–200 ng of gDNA and the VeriFi Hot Start Polymerase enzyme (PCR Biosystems Ltd., London, United Kingdom) in 50‐μL reactions. For protocol design, two PCR reactions were performed for each primer pair while one PCR reaction was performed for the haplotype diversity screen.

#### Gel electrophoresis

Entire PCR reactions (protocol design) or 5 μL of each PCR reaction (haplotype diversity) were mixed with Purple Gel Loading Dye (New England Biolabs, Ipswich, Massachusetts, USA) and analyzed by gel electrophoresis on a 1% (w/v) agarose gel containing RedSafe Nucleic Acid Staining Solution (iNtRON Biotechnology, Kirkland, Washington, USA) with Tris‐acetate‐EDTA (TAE) buffer. Gel images were captured on a Gel Doc XR+ Imaging System (Bio‐Rad, Hercules, California, USA).

### Multi‐locus amplicon sequencing using Oxford Nanopore Technologies (ONT)

#### Amplicon recovery and barcoding

PCR products were either recovered from agarose gels and cleaned using the Zymoclean Gel DNA Recovery Kit (Zymo Research, Irvine, California, USA) and then pooled per gDNA (protocol design) or directly pooled per gDNA (haplotype diversity) and cleaned using the DNA Clean & Concentrator‐5 Kit (Zymo Research). Amplicon pools were barcoded using the PCR‐free Native Barcoding Expansion kit (EXP‐NBD104; ONT, Oxford, United Kingdom) following the end‐preparation and barcoding instructions in the Ligation Sequencing Kit 109 (LSK‐SQK109; ONT) protocol.

#### Library preparation and sequencing

**Figure 1:**
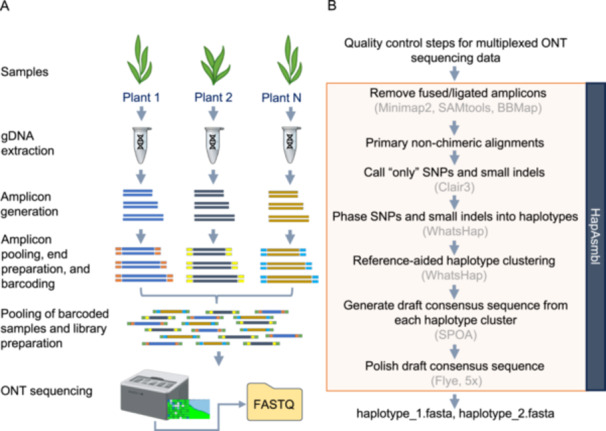
Overview of the multi‐locus Oxford Nanopore Technologies (ONT)–long‐read amplicon sequencing (LRAS) HapAsmbl pipeline. (A) The generation of multi‐locus LRAS data. Genomic DNA is extracted from leaf material of each sample and target‐gene amplicons are generated via PCR. Amplicons from each gDNA sample are pooled, end‐prepped, and barcoded with unique identifiers before all amplicons are combined and sequenced using ONT. (B) Haplotype‐aware amplicon assembly. Quality control steps recommended for ONT multiplexed data are performed prior to using the HapAsmbl pipeline. The HapAsmbl pipeline (orange box) begins with an additional quality control step to remove amplicons that are ligated together. Haplotypes are subsequently identified by calling and phasing only small nucleotide variants (i.e., SNPs and small indels). Reads from a haplotype are then clustered together with the aid of a reference sequence. After this, the consensus of each cluster is generated and polished. Text in gray notes the tools used.

Barcoded samples were pooled and prepared into sequencing libraries as per the “Ligation sequencing amplicons” protocol for the Ligation Sequencing Kit 110 (SQK‐LSK110; ONT). Depending on the experiment, 15–20 femtomole (fmol) of the library was loaded onto a FLO‐MIN106 (R9.4.1) Flow Cell on a GridION sequencer (ONT) and sequenced for 48–72 h. For haplotype analyses, three separate sequencing runs were performed using different R9.4.1 Flow Cells. This approach enabled the reuse of barcodes across multiple samples, as only 96 unique barcodes were available in the barcoding kit. Basecalling and demultiplexing were performed live with Guppy (v5.0.17 or higher; ONT) running on the MinKNOW software (v21.10.8 or higher; ONT) with a minimum Q‐score threshold of 9. All sequencing was conducted at the Otago Genomics Facility (OGF), University of Otago, New Zealand. We note that R9.4.1 flow‐cell chemistry has now been replaced with R10.4.1 chemistry, and that library preparation and sequencing adapted to R10.4.1 chemistry is suitable for the multi‐locus ONT‐LRAS approach outlined in Figure 1A.

#### Quality control prior to haplotype assembly

Quality control steps recommended for ONT data were performed before haplotype assembly (Figure S2A in Appendix S1) and should be selected based on the attributes of the dataset being used (e.g., R9.4.1 or R10.4.1 flow‐cell chemistry; see Mencius et al., 2025 for recommendations). For the ryegrass flowering gene dataset, post‐sequencing basecalling, demultiplexing, and adapter trimming were performed via command‐line using Guppy (v5.0.17 or higher; ONT) with the latest super‐accuracy basecalling model (dna_r9.4.1_450bps_sup.cfg). Chimeric reads containing within‐read adapters were split using Duplex tools v0.2.7 (https://github.com/nanoporetech/duplex-tools) with the ‐‐split_on_adapter parameter.

Residual terminal adapters were then removed. For our R9.4.1 datasets, these adapters were detected and removed using Porechop v2.04 (https://github.com/rrwick/Porechop). We note that this version of Porechop is no longer maintained and is a legacy tool. For applications requiring trimming of residual adapters from R10.4.1 LRAS data, the use of Porechop_ABI (Bonenfant et al., 2023) or alternative trimmers capable of auto‐detecting ONT adapters are recommended (Table S4 in Appendix S1).

Reads lower than a defined Q‐score and reads longer or shorter than expected target lengths were filtered using Filtlong v0.2.1 (https://github.com/rrwick/Filtlong). In this study, the mean sequencing quality score (‐‐min_mean_q) was used for quality filtering while the expected amplicon size ranges were used for read‐length filtering (‐‐min_length and ‐‐max_length). While R10.4.1 chemistry provides substantially improved raw read accuracy, our preliminary analyses indicate that chimeric reads and homopolymer‐related errors (albeit at a lower level) remain (data not shown). We therefore recommend applying equivalent, pre‐haplotype assembly quality control steps tailored to the chemistry used for ONT sequencing and the amplicon dataset (Mencius et al., 2025; Table S4 in Appendix S1).

### Haplotype assembly from ONT‐LRAS data

#### De novo haplotype assembly

Split (non‐chimeric) and filtered reads were mapped to alleles from the Kyuss ryegrass genome (Frei et al., 2021) with Minimap2 v2.23 (Li, 2018) using parameter ‐ax map‐ont, and unmapped reads were removed with SAMtools v1.15 (http://www.htslib.org/doc/1.15/samtools.html; Danecek et al., 2021). Identifiers of queries in the alignment file were used to subset the unfiltered FASTQ file with SeqKit v2.1.0 (Shen et al., 2016) using the grep ‐‐pattern‐file parameter. The subset of the FASTQ file was used for de novo (reference‐free) haplotype assembly with Flye v2.9 (Kolmogorov et al., 2019) using the parameters ‐‐nano‐raw, ‐‐meta, and ‐‐keep‐haplotypes. The parameter ‐‐min‐overlap 1000 was alternately included or excluded in different testing scenarios.

#### Reference‐aligned assembly

BAM files generated by SAMtools were used to call the full spectrum of genetic variants (SNPs, small indels, and structural variants) with Clair3 v1.05 or higher (Zheng et al., 2022) using the ‐‐enable_long_indel experimental parameter and other parameters recommended for amplicon sequence data. Small and long variants were subsequently phased into haplotypes using the whatshap phase in WhatsHap v1.2 (Martin et al., 2023), and the consensus sequence of haplotypes were generated using BCFtools v1.14 (https://github.com/samtools/bcftools; Danecek et al., 2021) with the consensus subcommand.

#### HapAsmbl: A custom pipeline for haplotype assembly

The custom pipeline (HapAsmbl; Figure 1B) begins by mapping FASTQ files to the reference sequence using Minimap2. As an extra quality control step, unmapped, non‐primary, and secondary alignments are then removed with SAMtools, after which concatenated or ligated amplicons (formed during library preparation) are filtered using BBMap v39.01 (https://sourceforge.net/projects/bbmap/) with the parameter clipfilter = 10 (Figure S2B in Appendix S1). For haplotype assembly, small variants (SNPs and indels) were called using Clair3 v1.05 or higher with the model r941_prom_sup_g5014 for R9.4.1 ONT data, which are then phased into haplotypes using WhatsHap v1.2. Reads from each haplotype were then clustered using whatshap haplotag and whatshap split in WhatsHap (Martin et al., 2023). Consensus sequences for each cluster were generated using SPOA v4.1.0 (Vaser et al., 2017) using the semi‐global alignment algorithm (‐‐algorithm −2). Each consensus was then polished iteratively over five rounds using Flye v2.9 (Kolmogorov et al., 2019) with the command flye polish ‐‐polish‐target‐‐iterations 5 along with ‐‐nano‐raw for uncorrected ONT reads. HapAsmbl has also been tested on and validated for haplotype‐aware assembly of R10.4.1 LRAS data, with chemistry‐specific parameter differences and recommendations between R9.4.1 and R10.4.1 detailed in Table S4 (Appendix S1). Code and instructions for running HapAsmbl on both R9.4.1 and R10.4.1 LRAS datasets are available on GitHub (https://github.com/eiwai81/HapAsmbl).

### Evaluation of haplotype‐aware assembly pipelines for ONT‐LRAS data

The *pVRN2A* region from F_2_ A24983 plants 10 (F10) and 12 (F12) were selected for the initial assessment of assembly pipelines. To establish a valid SNP–SV linkage reference for this comparison, *pVRN2A* amplicon reads of these samples were manually phased using the “Cluster (phase) alignments” experimental feature in IGV v2.16.2 (Thorvaldsdóttir et al., 2013) and assigned to either Haplotype 1 (H1) or 2 (H2) based on SNPs and two structural variants (~1300 bp and ~50 bp indels).

### Haplotype generation from the mitochondrial *cytochrome oxidase I (COI)* gene

The ability of the HapAsmbl pipeline to accurately recover two haplotypes from diploid heterozygous samples was validated using published *COI* amplicons (Maestri et al., 2019). Haploid ONT MinION data of four *COI* haplotypes (“BC01”, SRA no. SRR8986141; “BC03”, SRA no. SRR8986137; “BC05”, SRA no. SRR8986135; and “BC07”, SRA no. SRR8986147), along with their corresponding Sanger sequencing results (“BC01”, SRA no. SRR8986140; “BC03”, SRA no. SRR8986136; “BC05”, SRA no. SRR8986134; and “BC07”, SRA no. SRR8986146) were retrieved from the National Center for Biotechnology Information (NCBI) Sequence Read Archive (SRA). Different haploid *COI* ONT‐LRAS datasets with distinct alleles were combined into biallelic samples and assembled using HapAsmbl. Consensus sequences generated by HapAsmbl were compared to the Sanger sequencing reference using BLAST (Altschul et al., 1990) with default parameters and benchmarked based on overall percentage identity, number of mismatches, and number of gaps.

### Comparing haplotypes of ryegrass flowering genes from HapAsmbl and other technologies

Identification of unique alleles in the protocol design cohort was achieved through haplotype network maps constructed with PopART v1.7 (Leigh and Bryant, 2015) using multiple sequence alignments of HapAsmbl‐derived sequences generated with progressiveMauve (Darling et al., 2010) in Geneious (Dotmatics, Auckland, New Zealand). These unique haplotypes were compared to haplotypes assembled using bacterial artificial chromosome sequencing (BACS) and whole genome sequencing (WGS) to assess the accuracy of HapAsmbl‐derived sequences. *FT3* haplotypes (Skøt et al., 2011) and *VRN1* haplotypes (Asp et al., 2011) assembled via BACS were retrieved using GenBank accession numbers. For the *VRN1* BACS‐assembled data, an in silico PCR was performed using SeqKit v2.1.0, with *VRN1* primer sequences used for protocol development to provide a comparable region for evaluation. The WGS‐derived haplotypes were extracted from published reference genomes (Frei et al., 2021; Nagy et al., 2022).

BLAST (MegaBLAST) was used to compare BACS‐ and WGS‐derived haplotypes with those assembled using HapAsmbl with the parameters ‐evalue 1 × 10^−10^, ‐max_hsps 2, ‐max_target_seqs 1, and ‐perc_identity 99. BACS‐ and WGS‐derived haplotypes were also compared using BLAST to establish a baseline for expected sequence variation (SNPs and indels) between the two methods. Technological comparisons were based on (i) percentage identity, (ii) number of mismatches (used as proxy for SNPs), and (iii) number of gaps (used as proxy for indels). Percentage identities of 99.96–100%, 0 mismatches, and 0–1 gaps were interpreted as identical alleles.

Pairwise alignments between haplotypes derived from ONT‐LRAS, BACS, and WGS data and the reference set from the Kyuss genome (Frei et al., 2021) were generated using Minimap2 v2.23 and loaded onto IGV v2.16.2 to visually compare outputs from the three methods.

### Analysis of perennial ryegrass flowering‐related genes from the ONT‐LRAS‐HapAsmbl pipeline and other sources

Amplicons of *CO*, *FT3*, and *piVRN1* (Figure S1 and Table S3 in Appendix S1) were generated from 14 One^50^ and 141 F_2_ plants (Table S1 in Appendix S1) using ONT‐LRAS, and haplotypes were assembled using HapAsmbl. Unique gene haplotypes were determined using PopART v1.7 (Leigh and Bryant, 2015) as described above, and a node size threshold of three was applied to exclude singletons, rare alleles, and cryptic haplotypes. Representative sequences from each network node were aligned against the Kyuss v2 reference genome (Chen et al., 2024) using Minimap2 v2.23 and visualized in IGV v2.16.2 to assess the degree of sequence divergence.

To further explore allelic diversity at these three genes, multiple sequence alignments of all HapAsmbl‐derived sequences and published haplotypes from other ryegrass genotypes (Asp et al., 2011; Skøt et al., 2011; Frei et al., 2021; Nagy et al., 2022) were generated with progressiveMauve (Darling et al., 2010) and neighbor‐joining trees were constructed in Geneious. Gene trees were visualized and annotated using the Interactive Tree Of Life (iTOL) (Letunic and Bork, 2024).

## RESULTS

### Direct multi‐locus long‐read amplicon sequencing

To assist studies on gene diversity analysis and allele discovery in highly heterozygous plant populations, we aimed to develop an economical and high‐throughput method to generate sequences for the diploid haplotypes in individuals. We focused on perennial ryegrass and an ONT‐based multiplex LRAS approach that could be applied simultaneously to multiple loci (multi‐locus ONT‐LRAS), as outlined in Figure 1A.

To develop the multi‐locus ONT‐LRAS approach, we selected five flowering‐related genes (*FT3*, *FTL9*, *pVRN1*, *pVRN2A*, and *VRN2B*; Wang and Forster, 2017; Herridge et al., 2021) and used five plants from the cultivar One^50^ and five plants from the F_2_ population A24983 derived from a cross between the cultivar Stella and an Algerian accession (Table S1 in Appendix S1). gDNA was extracted from each plant along with a pooled sample containing leaf material from each of the five plants from each population to explore pooling as an efficient approach. Target regions of each gene were amplified by PCR (Figure S1 in Appendix S1). Gel electrophoresis confirmed amplification occurred in most samples, including some size variation indicative of allelic variation (Figure S3 in Appendix S1). There were low levels of some products, such as *VRN2B* in the F_2_ A24983 plants, but these were still used to help assess the level of product required for haplotype determination. The gel electrophoresis revealed drawbacks associated with pooling, as there were monomorphic amplicons for a target region when the individual plants showed clear size polymorphisms (see *pVRN2A* in F_2_ A29483 in Figure S3 in Appendix S1).

Amplicons from each gDNA sample were combined and barcoded using a PCR‐free approach, enabling an efficient workflow for generating multi‐locus ONT‐LRAS data (Figure 1A). The barcoded library was sequenced using ONT on a flow cell with other independent libraries. After basecalling with the super‐accuracy model, 331,044 reads were classified into one of the 12 barcodes used for the ryegrass amplicons. The total number of reads per barcoded sample ranged from 15,044 to 46,643, with the majority having a Q‐score of nine or higher (Figure S4A in Appendix S1), and the read length distribution formed distinct peaks corresponding to the expected lengths of the target amplicons (Figure S4B,C in Appendix S1). Additionally, subtle length variations were observed in certain amplicons across specific samples, consistent with size polymorphisms seen in the gel electrophoresis (Figure S3 in Appendix S1) and indicative of structural variants.

Quality control steps recommended for multiplexed ONT sequencing data, including splitting concatemers/chimeric amplicons containing within‐read adapters and filtering reads based on length and Q‐score, were applied prior to haplotype assembly (Figure S2A in Appendix S1). An additional, optional trimming step was used to remove residual terminal adapter sequences. Split and non‐chimeric filtered amplicons that did not map to any sequence in the reference set were also removed. At the end of this quality control workflow, a total of 104,418 filtered non‐chimeric reads were recovered (Figure S4D in Appendix S1). Despite the quality control steps, basecalling errors relating to size variation of homopolymer regions were still persistent in the dataset. These quality control steps were designed to be used with data generated with ONT R9.4.1 chemistry, and we recommend applying equivalent steps for R10.4.1 chemistry datasets (Table S4 in Appendix S1).

### Haplotype assembly using available tools with multi‐locus ONT‐LRAS data

For allelic diversity studies and allele discovery in plants with high levels of heterozygosity, amplicons generated via multi‐locus ONT‐LRAS must be accurately assembled to ensure an accurate sequence for each allele. To determine the best approach to achieve this, we initially evaluated two available haplotype‐aware assembly methods—a de novo assembly and a reference‐aligned assembly.

For the de novo assembly, the long‐read assembly program Flye (Kolmogorov et al., 2019) was trialed because it had a ‐‐keep‐haplotypes option to keep sequence haplotypes. The results produced by Flye varied considerably, depending on whether a minimum read overlap between reads in the LRAS data was specified (Figure S5 in Appendix S1; sequences provided in Appendix S2). Furthermore, there were cases in which either no sequence was assembled (*pVRN1* in plant O3) or more than two contigs were assembled from a diploid sample (*FT3* in F_2_ A24983 plants). Additionally, as with gel electrophoresis, the number of haplotypes in the pooled samples was often lower than the number identified in the individual plants.

The performance of the de novo assembly approach with Flye was also evaluated using the *pVRN2A* region of F_2_ A24983 plants 10 (F10) and 12 (F12), as gel electrophoresis of amplicons indicated these plants contained two distinct alleles (Figure S3 in Appendix S1) and the LRAS data of these plants could be manually phased and assigned to one of two haplotypes (H1 and H2) (Figure S6 in Appendix S1). Notably, a region containing six SNPs, followed by a ~1300‐bp and a ~50‐bp indel, could be used to distinguish these haplotypes. An assessment of this discriminative *pVRN2A* region in the assemblies of samples F10 and F12 generated by the de novo assembly method indicated that only the larger haplotype was assembled for these samples (Table 1). Given the substantial impact of parameters on the results generated by Flye and the high likelihood that alleles from a heterozygous plant would be missing in the assemblies, we concluded that a de novo assembly method was not ideally suited for highly heterozygous species.

**Table 1:** A comparison of the assembly results produced by three haplotype‐aware pipelines. The comparison shows a ~170‐bp region of the *VRN2A* promoter from two heterozygous diploid plants (F10 and F12; Figure S6 in Appendix S1) from the F_2_ A249843 population.^a^

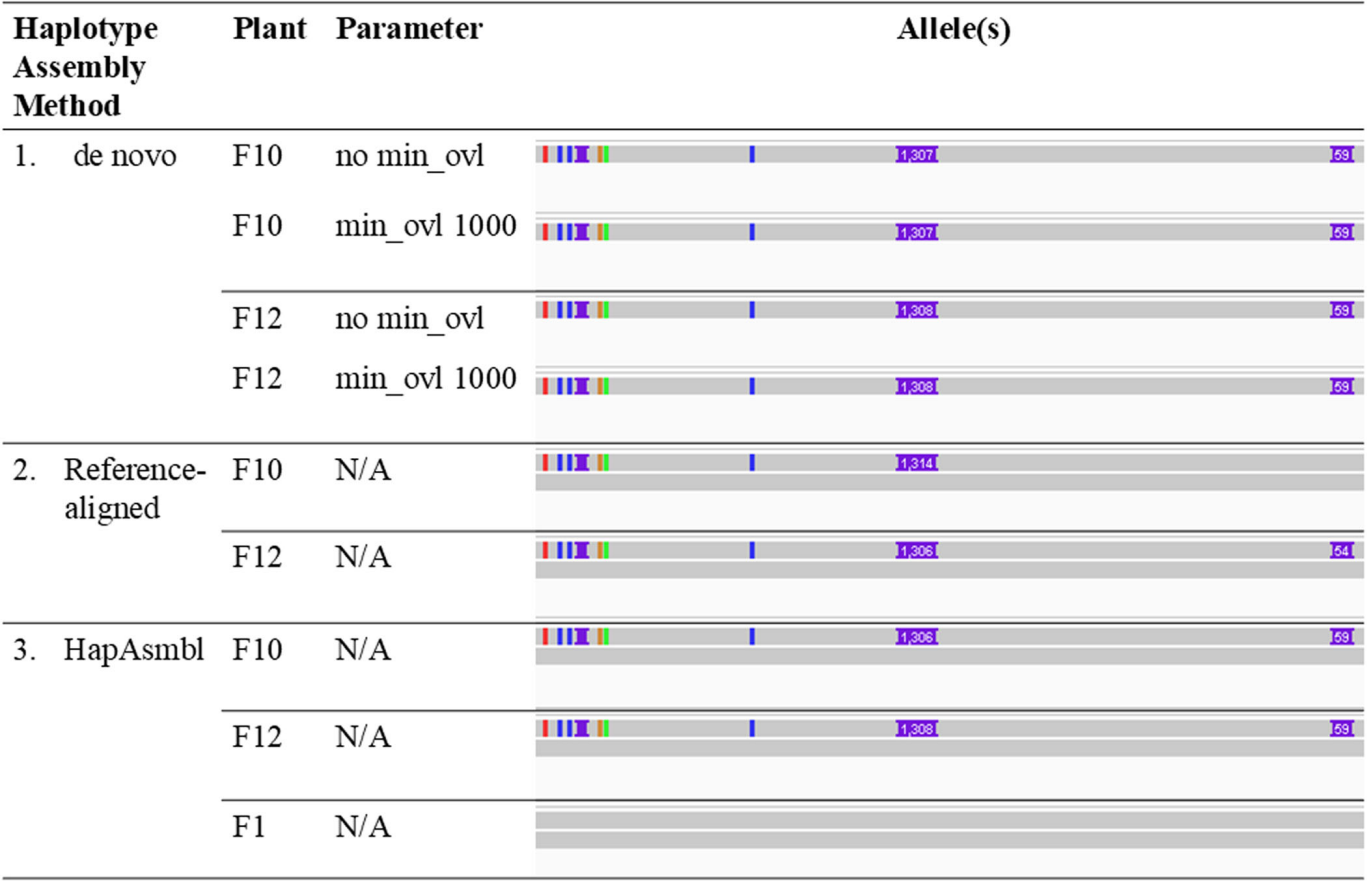

*Note*: min_ovl = minimum overlap between reads; N/A = not applicable.

^a^Gray bars represent sequences; colored lines represent variant bases relative to the allele in the Kyuss reference genome. Thick purple bars represent structural variants (~1300 bp and ~50 bp).

Next, we trialed a reference‐aligned approach using a Clair3‐WhatsHap‐BCFtools workflow. The Clair3 program was chosen for variant calling as this tool has been demonstrated to deliver comparatively higher indel calling performance and SNP detection accuracy with ONT sequencing data (Helal et al., 2022; Zheng et al., 2022; Hall et al., 2024) and because it had an experimental ‐‐enable_long_indel parameter for detecting structural variants, making it possible to call the full spectrum of variants in reads (https://github.com/HKU-BAL/Clair3). Additionally, WhatsHap was chosen for its ability to perform read‐based phasing of all types of variants, including small and large indels, into haplotypes (Martin et al., 2023). Using this pipeline, it was possible to retrieve two consensus sequences per target region for some individual samples (Figure S7 in Appendix S1; sequences provided in Appendix S3), but it was not possible to assemble any consensus sequence from the pooled samples, given that the Clair3 variant caller operates under the assumption that the input sequence data were derived from a diploid sample.

An examination of the discriminative *pVRN2A* region within the haplotypes generated for the F10 and F12 samples of F_2_ A24983 revealed that both the large and small alleles were present in the assemblies of both heterozygous samples (Table 1). However, the larger allele assembled for the F10 sample lacked the ~50‐bp indel. This observation suggested that this variant‐based haplotype assembly pipeline is able to handle the heterozygosity in ONT‐LRAS datasets, but runs the risk of assembling false positives if erroneous calls are made during the variant detection step.

### Development of the HapAsmbl pipeline for assembling haplotypes from multi‐locus LRAS data

Owing to the lack of tools or methods to accurately assemble sequence haplotypes in multi‐locus ONT‐LRAS data, a custom pipeline, HapAsmbl, was developed (Figure 1B). This pipeline is inspired by the logic of Phasebook (Luo et al., 2021) and combines existing software and elements from established pipelines. The HapAsmbl pipeline begins with an additional quality control step that removes fused/ligated amplicons formed during library preparation (Figures S2B, S8 in Appendix S1). When applied to the perennial ryegrass flowering‐time gene multi‐locus ONT‐LRAS data, the mean sequencing depth per gene following this step varied between approximately 3–7000× for the F_2_ A24983 samples and approximately 10–3500× for the One^50^ samples (Figure S9 in Appendix S1), broadly corresponding to the levels detected by gel electrophoresis (Figure S3 in Appendix S1).

Haplotypes in the resulting dataset are subsequently recovered by leveraging read‐based phasing to identify and cluster reads according to haplotype based on small nucleotide variants (SNPs and indels). A draft consensus sequence of each haplotype cluster is then generated and polished. Using this pipeline, we were able to recover two consensus sequence haplotypes from a heterozygous diploid individual, as evidenced by the two *pVRN2A* haplotypes in the assemblies of plants F10 and F12 (Table 1). Moreover, the pattern of small and large variants in the two assembled haplotypes matched those in the raw ONT‐LRAS data.

In instances where all reads originate from a single haplotype, implying potential homozygosity, the protocol was designed to assemble two similar consensus sequences from the F_2_ plants (e.g., *pVRN2A* of the F1 plant; Table 1). As such, two sequences were assembled for each target gene from the multi‐locus LRAS data of each diploid plant, irrespective of sample zygosity, even for low‐abundance amplicons (e.g., *VRN2B* amplicons in the F_2_ A24983 plants). Using the results from the HapAsmbl pipeline, we were also able to establish the putative diploid genotype for each plant from the F_2_ A24983 and One^50^ perennial ryegrass populations and to infer their putative zygosities at each locus (Figure S10 in Appendix S1). As noted with the Clair3‐WhatsHap‐BCFtools workflow, HapAsmbl could not be applied to the LRAS data from pooled samples due to the current limitation of Clair3, which does not support sequence data of samples with a ploidy level exceeding two. Given that R9.4.1 chemistry has now been updated, we have adapted and validated (data not shown) the HapAsmbl pipeline for use with R10.4.1 chemistry and provide an option for R10.4.1 amplicon datasets on the project's GitHub repository.

### Validation of the HapAsmbl pipeline using verified *COI* LRAS data

Following the development of the HapAsmbl pipeline, we wanted to use independent data to validate its ability to assemble two haplotypes from a heterozygous ONT amplicon dataset. As most published ONT amplicon datasets are from haploid or homozygous diploid samples, we used the ~700‐bp MinION amplicon sequences of seven haploid samples (BC01–BC07) of the mitochondrial *COI* gene from two snails and five beetles that had been confirmed by Sanger sequencing (Maestri et al., 2019). To simulate heterozygosity, haploid ONT data of four samples were combined into six mixed samples (BC01_BC03, BC01_BC05, BC01_BC07, BC03_BC05, BC03_BC07, and BC05_BC07) featuring distinct biallelic combinations.

**Figure 2:**
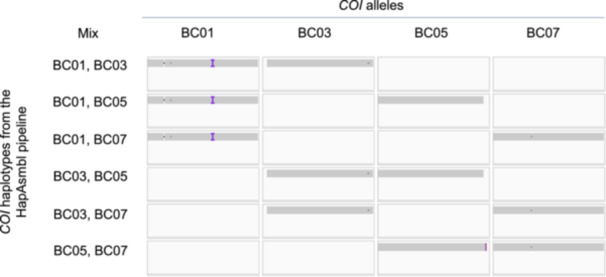
Comparison of a ~700‐bp region of the mitochondrial *cytochrome oxidase I* (*COI*) gene assembled from six mixed heterozygous samples using HapAsmbl. Column labels denote barcode identifiers corresponding to unique *COI* alleles based on Sanger sequencing as reported by Maestri et al. (2019). Panels show pairwise alignments between unique alleles assembled from six mixed samples simulating heterozygosity using the HapAsmbl pipeline and the Sanger reference set. Gray bars represent a sequence, thin purple bars indicate small insertions relative to the Sanger reference sequence, and gaps correspond to small deletions relative to the Sanger reference sequence. Performance of HapAsmbl was assessed based on the number of recovered *COI* haplotypes from each mixed sample and on the profile of SNPs and indels of haplotypes relative to the Sanger reference allele in each pairwise alignment.

When HapAsmbl‐derived *COI* sequences were aligned to a reference set of the four samples obtained through Sanger sequencing, all expected alleles across the six biallelic mixtures were recovered (Figure 2). Additionally, the average sequence similarity for HapAsmbl‐derived sequences corresponding to each *COI* allele closely matched those of the consensus sequences generated by the ONTrack pipeline used by Maestri et al. (2019). BLAST comparisons revealed no mismatches between HapAsmbl sequences and the sequences determined by Sanger sequencing, although one to three gaps were present in sequences assembled using HapAsmbl, giving overall sequence similarities of 99.54% to 100% (Figure 2, Table 2). These gaps were attributed to persistent homopolymer errors in certain HapAsmbl contigs. Similar indel errors in homopolymer regions were documented in the original study by Maestri et al. (2019). Overall, analysis of the *COI* data indicated that HapAsmbl can reliably recover unique alleles and haplotypes from heterozygous samples.

**Table 2:** Performance metrics of the HapAsmbl pipeline using the *COI* gene from Nanopore amplicon sequences.^a^

Sanger sequence ID (original paper)	Mixed sample ID (HapAsmbl contig matching Sanger sequence)	Length (HapAsmbl)^b^	Mismatches (HapAsmbl)^b^	Gaps (HapAsmbl)^b^	Identical matches (%) (HapAsmbl)^b^
BC01	BC01_BC03 (h1)	653	0	3	99.54
	BC01_BC05 (h2)	653	0	3	99.54
	BC01_BC07 (h1)	653	0	3	99.54
BC03	BC01_BC03 (h2)	663	0	1	99.85
	BC03_BC05 (h2)	663	0	1	99.85
	BC03_BC07 (h2)	663	0	1	99.85
BC05	BC01_BC05 (h1)	674	0	0	100
	BC03_BC05 (h1)	674	0	0	100
	BC05_BC07 (h1)	688	0	0	100
BC07	BC01_BC07 (h2)	536	0	1	99.81
	BC03_BC07 (h1)	536	0	1	99.81
	BC05_BC07 (h2)	536	0	1	99.81

^a^ The *COI* Nanopore dataset and Sanger reference sequences used for benchmarking were published by Maestri et al. (2019). Benchmarking was performed by querying each HapAsmbl‐derived *COI* haplotype against its corresponding Sanger reference sequence using BLAST. Performance of HapAsmbl was based on three alignment metrics: (i) percentage identity, (ii) number of mismatches, and (iii) number of gaps.

^b^ Comparison relative to the sequence generated for the sample via Sanger sequencing by Maestri et al. (2019).

### HapAsmbl provides known and new haplotypes of ryegrass flowering‐related genes

**Figure 3:**
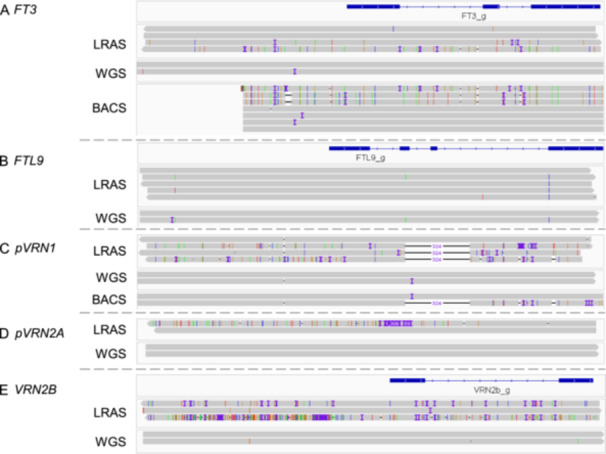
Comparison of five floral gene alleles generated via different sequencing technologies. Alignment of alleles of *FT3* (A), *FTL9* (B), *pVRN1* (C), *pVRN2A* (D), and *VRN2B* (E) from the ONT‐LRAS HapAsmbl pipeline (LRAS), whole genome sequencing (WGS) of the Kyuss and P226/135/16 genotypes (Frei et al., 2021; Nagy et al., 2022), and bacterial artificial chromosome sequencing (BACS) (Asp et al., 2011; Skøt et al., 2011). Gray bars represent an allele, while colored lines indicate variant bases relative to the Kyuss reference allele. Structural variations are denoted by thick purple bars or black lines within gray bars, with numbers indicating the size of the variation. Gene models in the 5′ to 3′ direction are shown in the top panel of the different alignments, with the thick blue bar representing exons and thin blue lines representing introns. Comparison of alleles across technologies was based on the profile of SNPs, indels, and structural variants relative to the reference allele in each pairwise alignment.

To confirm the utility of using HapAsmbl to detect haplotype variation in a highly heterozygous species, we compared the haplotypes of ryegrass flowering‐related genes from the ONT‐LRAS‐HapAsmbl workflow with sequences determined by established methods. We first identified unique alleles for the five genes in the F_2_ A24983 and One^50^ plants from HapAsmbl (Figure S10 in Appendix S1; sequences provided in Appendix S4) and aligned them to sequences from WGS (Frei et al., 2021; Nagy et al., 2022) and BACS (Asp et al., 2011; Skøt et al., 2011) (Figure 3). The comparison revealed that some of the haplotypes identified in the ONT‐LRAS‐HapAsmbl workflow corresponded to alleles generated with these traditional methods, with ≥99.96% identity, 0 mismatches, and 0–1 gaps (Table 3). In addition, the consensus sequences assembled from LRAS data using HapAsmbl were comparable to high‐quality assemblies obtained through BACS and WGS, sharing similar profiles of SNPs (0) and indels (1–7) when compared to the baseline established with the BACS‐ and WGS‐derived sequences (0 SNPs, 1–8 indels) (Figure 3, Table 3). Moreover, the analysis revealed the presence of novel alleles in the 10 plants used for protocol development that have not been previously documented, as only six of the 18 unique haplotypes from these plants shared at least 99.96% identity with previously published sequences (Table 3). These findings demonstrate that the ONT‐LRAS‐HapAsmbl workflow offers a direct, efficient, and cost‐effective method for sequencing amplicons derived from heterozygous samples.

**Table 3:** Comparison of sequence similarity among floral gene haplotypes derived from whole genome sequencing (WGS), bacterial artificial chromosome sequencing (BACS), and the long‐read amplicon sequencing (LRAS) HapAsmbl pipeline. Comparisons across technologies were based on three alignment metrics: (i) percentage identity, (ii) number of mismatches (used as a proxy for SNPs), and (iii) number of gaps (used as a proxy for indels).

Query^a^^,^ b ^,^ c	Subject^a^^,^ b ^,^ c	Alignment length	Identity (%)	Mismatches	Gaps
BLAST alignment statistics of BACS or WGS alleles against LRAS haplotypes					
VRN2A_kyus^a^	barcode01.VRN2A.h1^c^	2425	100	0	0
VRN2A_nagy^a^	barcode01.VRN2A.h1^c^	2425	100	0	0
FT3_kyus^a^	barcode01.FT3.h1^c^	1744	100	0	0
FTL9_nagy^a^	barcode07.FTL9.h1^c^	2796	99.96	0	1
FTL9_kyus^a^	barcode07.FTL9.h1^c^	2795	99.89	3	0
VRN2B_nagy^a^	barcode01.VRN2B.h1^c^	4126	99.81	2	6
VRN2B_kyus^a^	barcode01.VRN2B.h1^c^	4126	99.81	3	5
VRN1_nagy^a^	barcode03.VRN1.h1^c^	2252	99.78	0	5
^a^VRN1_kyus^a^	barcode03.VRN1.h1^c^	2253	99.73	0	6
FT3_nagy^a^	barcode01.FT3.h1^c^	1750	99.66	0	6
VRN1_falster^b^	barcode03.VRN1.h1^c^	1355	100	0	0
FN993918_B^b^	barcode01.FT3.h1^c^	1360	100	0	0
FN993922_F^b^	barcode01.FT3.h1^c^	1360	99.85	0	2
FN993920_D^b^	barcode01.FT3.h1^c^	1362	99.85	0	2
FN993921_E^b^	barcode01.FT3.h2^c^	1404	99.79	1	2
VRN1_veyo^b^	barcode03.VRN1.h1^c^	2254	99.69	0	7
FN993919_C^b^	barcode01.FT3.h1^c^	1366	99.56	0	6
BLAST alignment statistics of alleles assembled from BACS against alleles from WGS					
FN993918_B^b^	FT3_kyus^a^	1393	100	0	0
FN993919_C^b^	FT3_nagy^a^	1399	100	0	0
VRN1_veyo^b^	VRN1_kyus^a^	2285	99.96	0	1
VRN1_falster^b^	VRN1_nagy^a^	1375	99.93	0	1
VRN1_veyo^b^	VRN1_nagy^a^	2284	99.91	0	2
FN993920_D^b^	FT3_kyus^a^	1395	99.86	0	2
FN993922_F^b^	FT3_kyus^a^	1393	99.86	0	2
VRN1_falster^b^	VRN1_kyus^a^	1377	99.78	0	3
FN993918_B^b^	FT3_nagy^a^	1399	99.57	0	6
FN993919_C^b^	FT3_kyus^a^	1399	99.57	0	6
FN993920_D^b^	FT3_nagy^a^	1401	99.43	0	8
FN993922_F^b^	FT3_nagy^a^	1399	99.43	0	8

^a^ Haplotypes of *FT3* and *VRN1* extracted from assemblies of BACS data (Asp et al., 2011; Skøt et al., 2011).

^b^ Haplotypes of *FT3*, *FTL9*, *pVRN1*, *pVRN2a*, and *VRN2B* extracted from the Kyuss and P226/135/16 reference genomes assembled from WGS data (Frei et al., 2021; Nagy et al., 2022).

^c^ Unique haplotypes of the same five floral genes among alleles assembled from LRAS data of 10 F_2_ A24983 and One^50^ ryegrass plants.

### HapAsmbl can be used to survey allelic diversity within diverse populations

**Figure 4:**
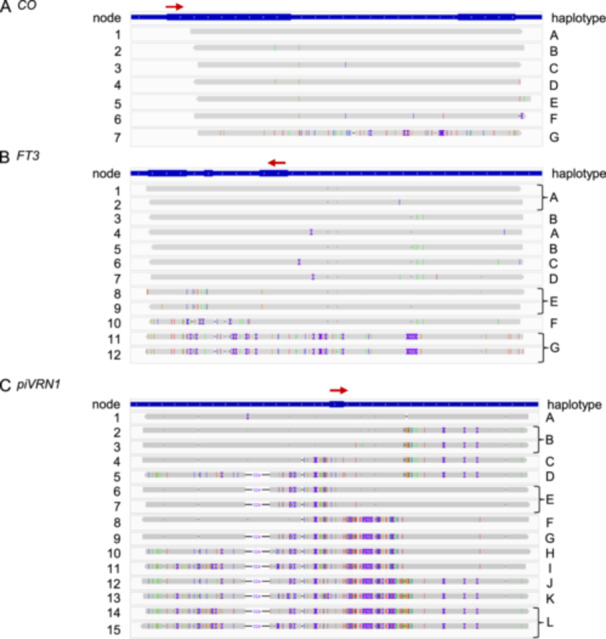
Predominant haplotypes of three floral genes in the eight F_2_ ryegrass populations surveyed in this study. Representative sequences of the unique alleles of *CO* (A), *FT3* (B), and *piVRN1* (C) mapped to the Kyuss v2 reference genome (Chen et al., 2024). Nodes are indicated on the left, with similar nodes collapsed into haplotypes indicated on the right. The window size of the gene region analyzed is ~2.1 kbp for *CO* and *FT3* and 4.5 kbp for *piVRN1*. Gene models are shown in the top panel of the different alignments, with the thick blue bar representing exons, thin blue lines showing introns, and red arrows indicating the first exon and the orientation of the gene. Colored lines represent variant bases relative to the Kyuss allele. Structural variations are indicated by thick purple bars or black lines within gray bars. Haplotypes were compared and collapsed based on the profile of SNPs, indels, and structural variants relative to the reference allele in each pairwise alignment.

A key motivation for our development of the ONT‐LRAS‐HapAsmbl pipeline was to assemble a greater number of haplotypes from large sample sizes in a single sequencing experiment than is feasible with WGS and BACS. We therefore tested the scalability of the mutli‐locus ONT‐LRAS‐HapAsmbl workflow by surveying the predominant gene haplotypes of three flowering‐related genes, *CO*, *FT3*, and *VRN1* (using the *piVRN1* region; Figure S1 in Appendix S1), in 141 ryegrass plants sampled from eight genetically diverse F_2_ populations (Table S1 in Appendix S1). Clustering of the ONT‐LRAS‐HapAsmbl–derived sequences (~282 sequences per gene), using haplotype network analysis and ranking nodes by size, identified seven *CO*, 12 *FT3*, and 15 *piVRN1* nodes, each representing a unique haplotype, with varying levels of abundance (Figure S11 in Appendix S1; sequences provided in Appendices S5–S7). Pairwise alignment of the representative sequence from each node to the Kyuss v2 reference genome (Chen et al., 2024) was used to manually resolve redundant or highly similar haplotypes, resulting in consolidation of the 12 *FT3* nodes into seven predominant haplotypes (haplotypes A to G) and the 15 *piVRN1* nodes into 12 predominant haplotypes (haplotypes A to L; Figure 4). The number of putative haplotypes inferred per gene via HapAsmbl falls within the range of the haplotype diversity reported for other genes in perennial ryegrass (Ponting et al., 2007; Skøt et al., 2007; Xing et al., 2007; Brazauskas et al., 2010; Fiil et al., 2011).

The alignments also revealed varying degrees of sequence divergence among gene haplotypes relative to the reference Kyuss allele. For instance, *CO* haplotypes had low divergence from the reference allele, differing by only a few SNPs, except for the haplotype G (sequence at node 7; Figure 4A). On the other hand, haplotypes of *FT3* showed a moderate degree of divergence from the reference allele, with haplotype G (nodes 4 and 5) showing higher levels of genetic variation than other haplotypes (Figure 4B). The *piVRN1* haplotypes exhibited the highest level of variability and sequence divergence from the Kyuss reference allele; this was attributed to numerous small and large mutations, including multiple SNPs, indels, a ~300‐bp deletion in the promoter region, and a ~200‐bp insertion in the first intron (Figure 4C).

### Discovering novel alleles with the ONT‐LRAS‐HapAsmbl pipeline

To further explore allelic variation in *FT3* and *piVRN1* and demonstrate the potential of the ONT‐LRAS‐HapAsmbl workflow for the rapid discovery of novel alleles, we compared *FT3* and *piVRN1* sequences from the F_2_ and One^50^ ryegrass populations to previously reported alleles.

**Figure 5:**
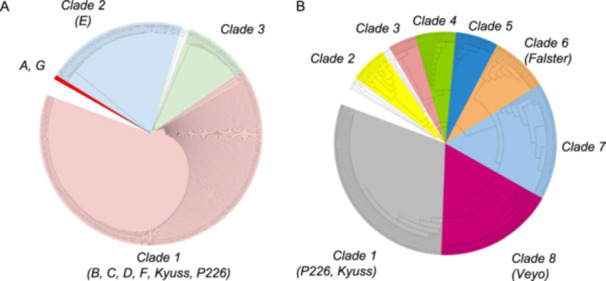
Phylogenetic analysis of *FT3* and *piVRN1* sequences in different ryegrass genotypes. (A) A neighbor‐joining tree of 319 *FT3* alleles comprising the seven haplotypes (A–G) published by Skøt et al. (2011), alleles from the Kyuss and P226/135/16 (P226) genotypes (Byrne et al., 2015; Frei et al., 2021; Nagy et al., 2022), 282 alleles from 141 F_2_ plants (this study), and 28 alleles from 14 One^50^ plants (this study). The tree delineates three major clades: Clade 1 comprises F_2_ and One^50^ alleles, as well as the published haplotypes B, C, D, and F (Skøt et al., 2011), along with sequences from the reference genomes; Clade 2 contains the E haplotype (Skøt et al., 2011) and alleles from One^50^ and F_2_ plants; and Clade 3 consists mainly of sequences from F_2_ and One^50^ plants. (B) A phylogenetic tree of the *piVRN1* region based on a ~4.5‐kbp region in 314 alleles assayed from eight F_2_ (282 alleles) and One^50^ (24 alleles) populations, as well as published alleles from the Falster, Veyo, Kyuss, and P226/135/16 genotypes (Asp et al., 2011; Frei et al., 2021; Nagy et al., 2022). The *piVRN1* sequences form eight major clades, with Clade 1 (P226, Kyuss), Clade 6 (Falster), and Clade 8 (Veyo) containing published sequences alongside F_2_ and One^50^ alleles, while Clades 2–5 and 7 comprise alleles shared between just F_2_ and One^50^ plants. *piVRN1* alleles outside the main clades likely represent singletons, rare alleles, or low‐frequency haplotypes within the F_2_ populations.

The *FT3* dataset (*n* = 319) included 282 alleles from 141 F_2_ plants (described above) and 28 alleles from 14 One^50^ plants assembled using the ONT‐LRAS‐HapAsmbl workflow, along with seven haplotypes (A–G) reported by Skøt et al. (2011) and alleles from reference genomes of Kyuss and P226/135/16 (Frei et al., 2021; Nagy et al., 2022). Phylogenetic analysis of this collection revealed three primary clades (Figure 5A). Clade 1 contained the most sequences from the F_2_ populations and One^50^, along with four haplotypes (B–D, F) published by Skøt et al. (2011) and alleles from the reference genomes. Haplotypes A and G from Skøt et al. (2011) (shown in red in Figure 5A) clustered separately and were absent in the F_2_ and One^50^ populations. Clade 2 contained haplotype E from Skøt et al. (2011), along with several alleles from F_2_ and One^50^ plants. Clade 3 was composed entirely of *FT3* sequences from the F_2_ populations and One^50^ plants, suggesting the presence of an undescribed allele in these populations.

For *piVRN1*, the dataset (*n* = 314) included 310 alleles from the 141 F_2_ and 14 One^50^ plants, along with published alleles from Falster, Veyo, Kyuss, and P226/135/16 ryegrass genotypes (Asp et al., 2011; Frei et al., 2021; Nagy et al., 2022). These alleles clustered into eight major clades (Figure 5B). The majority of *piVRN1* alleles from the F_2_ and One^50^ plants were grouped alongside alleles from P226/135/16 and Kyuss (Clade 1), Falster (Clade 6), or Veyo (Clade 8). Clades 2–5 and 7 consisted of alleles that were exclusively shared between the F_2_ and One^50^ plants, suggesting the presence of five previously undescribed *VRN1* alleles in these populations. A few *piVRN1* alleles were also found outside the main clades, likely representing singletons, rare alleles, or low‐frequency haplotypes within the F_2_ populations.

## DISCUSSION

The ability to pair barcoding with long‐read sequencing technologies, such as ONT, has enabled the direct end‐to‐end sequencing of long DNA molecules in an accessible and economic manner. This capability has driven the growing adoption of LRAS across diverse applications, including microbiome profiling and ecological diversity studies based on marker genes such as *COI* and 16S rRNA (Benítez‐Páez and Sanz, 2017; Maestri et al., 2019; Deep et al., 2023; Zhang et al., 2023b; Schacksen et al., 2024), the detection of mutations generated by site‐directed mutagenesis or gene editing using CRISPR‐Cas technologies (McCabe et al., 2024), and medical diagnostics (Watson et al., 2021; Charnaud et al., 2022; Whitford et al., 2022; Framst et al., 2024). LRAS also provides an opportunity to uncover allelic variation in plants (Makhoul et al., 2022; Polkhovskaya et al., 2024). In this study, we have paired multi‐locus LRAS using ONT sequencing with HapAsmbl, a reference‐aided pipeline for assembling haplotypes, providing a useful tool for surveying allelic variation, particularly for outcrossing species with extensive genetic and allelic variation, making it useful for a range of plant species.

This multi‐locus LRAS HapAsmbl pipeline presents several distinct benefits. By balancing speed, user‐friendliness, and high‐resolution information, the multi‐locus ONT‐LRAS‐HapAsmbl protocol provides haplotype data within target amplicons at levels comparable to those obtained via amplicon cloning and sequencing, but at reduced cost and time. This makes it ideal for population‐scale studies. While SRAS can also be applied at a population scale, leveraging long‐read data and HapAsmbl overcomes limitations experienced with SRAS, enabling the generation of more contiguous, phased haplotypes and efficiently handling multi‐locus data across large populations. The modular reference‐aided design of HapAsmbl also allows it to scale efficiently for large population samples and an expanded range of gene targets, making it adaptable to larger studies, which is valuable for breeding programs that rely on rapid and accurate haplotype analysis.

LRAS technologies, particularly ONT, are susceptible to several errors that can hinder accurate haplotype assembly from amplicon data. This includes concatenated reads containing within‐read adapters and ligated/fused amplicons that are introduced during library preparation, as well as sequencing errors in homopolymer regions. The HapAsmbl protocol mitigates these through meticulous quality control, read‐based phasing, and iterative polishing. Although some variation in homopolymer regions may remain in the final assemblies, especially when working with ONT‐LRAS data generated from older flow cell chemistries and basecallers, this does not impact the overall SNP‐indel‐SV linkage structure of the resulting haplotypes. Moreover, ongoing improvements to flow‐cell chemistry will further improve haplotype resolution quality and fidelity of haplotype information generated via HapAsmbl, making it more robust for applications requiring high accuracy, such as genomic selection and marker‐assisted breeding.

HapAsmbl is particularly useful for any highly polymorphic plant species that would benefit from haplotype‐aware genomic analyses. Our survey of the genetic diversity of three flowering‐related genes across several populations of perennial ryegrass demonstrated how the multi‐locus ONT‐LRAS‐HapAsmbl workflow can be integrated into haplotype analysis using clustering tools like PopART (Leigh and Bryant, 2015). Notably, for *FT3* and *VRN1*, the assembled haplotypes included those identified using other approaches, including BACS (Asp et al., 2011; Skøt et al., 2011) and WGS (Frei et al., 2021; Nagy et al., 2022), along with novel alleles, highlighting the high‐throughput enabled by the multi‐locus ONT‐LRAS‐HapAsmbl workflow. Given the key role of *FT3* and *VRN1* in flowering (Asp et al., 2011; Skøt et al., 2011; Wang and Forster, 2017) and the agronomic importance of flowering (heading date) in perennial ryegrass (Herridge et al., 2021), the addition of these haplotypes to the pool of known sequences will also help inform research in this area. In future work, haplotypes generated for genes such as *CO*, *FT3*, and *VRN1* could be compared to phenotypic data from larger sample sizes to identify alleles associated with different flowering responses or other key traits.

In conclusion, HapAsmbl offers a practical and efficient solution for haplotype assembly in genetically diverse populations and is applicable across a wide range of taxa. Its robustness in outcrossing and highly polymorphic species, together with its scalability for large cohorts, makes it a valuable tool for population genomics, evolutionary studies, applied breeding, and conservation programs, as more plant genomes become available.

## AUTHOR CONTRIBUTIONS

A.O.F., A.C., R.P.H., R.C.M., and L.R.B. conceptualized and designed the multi‐locus ONT‐LRAS workflow. A.O.F. and A.C. designed and performed the ONT sequencing experiments. A.O.F. performed the bioinformatics and data analysis. A.O.F. and L.R.B. drafted the manuscript. All authors revised, edited, and approved the final manuscript.

## Supporting information


**Appendix S1**: Supplemental tables and figures for “HapAsmbl: A reference‐aided pipeline for assembling haplotypes in Nanopore amplicon sequence data of polymorphic populations.”
**Figure S1**: Diagrammatic overview of regions in five perennial ryegrass flowering‐time genes assayed using long‐read amplicon sequencing.
**Figure S2**: Quality control steps. (A) Workflow for post‐sequencing base calling, read quality control, and filtering applied to Oxford Nanopore Technologies (ONT)‐based long‐read amplicon sequencing (LRAS) data prior to haplotype assembly. (B) Additional quality control steps incorporated into the HapAsmbl pipeline to remove concatenated amplicons.
**Figure S3**: Gel electrophoresis of the PCR products of five ryegrass flowering‐time genes (*FT3*, *FTL9*, *pVRN1*, *pVRN2A*, *VRN2B*) from gDNA from five plants from the F_2_ A24983 population (plants F1, F9, F10, F12, F18, and a pooled sample) and One^50^ (plants O1, O2, O3, O5, O11, and a pooled sample).
**Figure S4**: Key results of amplicon sequencing of ryegrass flowering genes using ONT sequencing.
**Figure S5**: Results of haplotype assembly using the de novo method. The figure shows the total number of sequences assembled from amplicon sequences of each target gene (right) for each sample using Flye's de novo assembly algorithm. The column name (top) indicates the run parameter: meta specifies that data has uneven coverage, while min_ovl indicates a minimum read overlap of 1000 bp among reads. Six gDNA samples (five individual plants and one pooled) were each prepared for two perennial ryegrass populations, F_2_ A249843 and One^50^. Plants used in preparing individual samples were diploid (maximum of two alleles). The pooled gDNA sample was prepared from pooled leaf materials of the five individual plants. Sequences are provided in Appendix S2.
**Figure S6**: Genetic and haplotype variation within a ~900‐bp region of the VRN2A promoter of the F10 plant.
**Figure S7**: Results of the reference‐aligned haplotype assembly using a Clair3‐WhatsHap‐BCFtools workflow. Row labels represent sample names while column labels represent target genes. Assemblies of each sample have been mapped to the allele of the corresponding gene from the Kyuss reference genome (Frei et al., 2021). Gray bars represent sequences. Colored lines represent variant bases relative to the allele in the Kyuss reference genome. Thick purple bars represent structural variants. The identity of genes (pVRN1 and VRN2B) with a high number of differences to the reference genes was confirmed by BLAST. Haplotype sequences are provided in Appendix S3.
**Figure S8**: Impact of filtering of ligated amplicons with BBMap prior to haplotype assembly.
**Figure S9**: The mean depth of sequencing of each target amplicon for samples in F_2_ A24983 (A) and One^50^ (B) ryegrass populations after removing chimeric sequences in the HapAsmbl pipeline.
**Figure S10:** Results from the HapAsmbl pipeline. Row labels represent sample names, while column labels represent target genes. Assemblies of each sample have been mapped to the allele of the corresponding gene from the Kyuss reference genome (Frei et al., 2021). Gray bars represent sequences, colored lines represent variant bases relative to the allele in the Kyuss reference genome, and thick purple bars represent structural variants. The identity of genes (*pVRN1* and *VRN2B*) with a high number of differences to the reference genes was confirmed by BLAST. Haplotype sequences are provided in Appendix S4.
**Figure S11:** Distribution of unique sequences (nodes) across three floral genes—*CO* (A), *FT3* (B), and *VRN1* (C)—in 141 F_2_ perennial ryegrass plants.
**Table S1.** Perennial ryegrass germplasm used in this study.
**Table S2**: Gene identifiers for genes targeted for amplicon sequencing. Identifiers are based on the Kyuss_2.0 reference genome (Chen et al., 2024).
**Table S3**: Sequences of gene‐specific primers targeting regions within six perennial ryegrass flowering‐time genes used in protocol design and haplotype diversity studies.
**Table S4.** Comparison of tools for quality control and HapAsmbl for ONT R9.4.1 and 10.4.1 flow cell chemistries.

**Appendix S2**: pipeline1.denovo.fasta: Haplotypes assembled using pipeline 1 in protocol design.

**Appendix S3**: pipeline2_ref_guided.fasta: Haplotypes assembled using pipeline 2 in protocol design.

**Appendix S4**: pipeline3_hapasmbl.fasta: Haplotypes assembled using HapAsmbl in protocol design.

**Appendix S5**: popart_FT3_nodes_f2s.fasta: FT3 nodes from the haplotype diversity screen.

**Appendix S6**: popart_piVRN1_nodes_f2s.fasta: VRN1 nodes from the haplotype diversity screen.

**Appendix S7**: popart_CO_nodes_f2s.fasta: CO nodes from the haplotype diversity screen (Figure 4).

## Data Availability

All assemblies generated in this article are available in the accompanying online Supporting Information. The FASTQ files of the Nanopore amplicon sequencing data underlying this article are available from the National Center for Biotechnology Information Sequence Read Archive (SRA) with the BioProject accession number PRJNA1419901. All codes and step‐by‐step instructions for HapAsmbl are available on GitHub (https://github.com/eiwai81/HapAsmbl).
