# Supplementary material for: HapAsmbl: A reference‐aided pipeline for assembling haplotypes in Nanopore amplicon sequence data of polymorphic populations
Source: Appl Plant Sci. 2026 Jun 12;14(3):e70062. doi: 10.1002/aps3.70062 (PMC13287972; doi:10.1002/aps3.70062)
Supplement: Supplementary file 1 — Appendix S1: Supplemental tables and figures for “HapAsmbl: A reference‐aided pipeline for assembling haplotypes in Nanopore amplicon sequence data of polymorphic populations.” Figure S1: Diagrammatic overview of regions in five perennial ryegrass flowering‐time genes assayed using long‐read amplicon sequencing. Figure S2: Quality control steps. (A) Workflow for post‐sequencing base calling, read quality control, and filtering applied to Oxford Nanopore Technologies (ONT)‐based long‐read amplicon sequencing (LRAS) data prior to haplotype assembly. (B) Additional quality control steps incorporated into the HapAsmbl pipeline to remove concatenated amplicons. Figure S3: Gel electrophoresis of the PCR products of five ryegrass flowering‐time genes (FT3, FTL9, pVRN1, pVRN2A, VRN2B) from gDNA from five plants from the F2 A24983 population (plants F1, F9, F10, F12, F18, and a pooled sample) and One50 (plants O1, O2, O3, O5, O11, and a pooled sample). Figure S4: Key results of amplicon sequencing of ryegrass flowering genes using ONT sequencing. Figure S5: Results of haplotype assembly using the de novo method. The figure shows the total number of sequences assembled from amplicon sequences of each target gene (right) for each sample using Flye's de novo assembly algorithm. The column name (top) indicates the run parameter: meta specifies that data has uneven coverage, while min_ovl indicates a minimum read overlap of 1000 bp among reads. Six gDNA samples (five individual plants and one pooled) were each prepared for two perennial ryegrass populations, F2 A249843 and One50. Plants used in preparing individual samples were diploid (maximum of two alleles). The pooled gDNA sample was prepared from pooled leaf materials of the five individual plants. Sequences are provided in Appendix S2. Figure S6: Genetic and haplotype variation within a ~900‐bp region of the VRN2A promoter of the F10 plant. Figure S7: Results of the reference‐aligned haplotype assembly using a Clair3‐WhatsH [file APS3-14-e70062-s003.docx]

## **Appendix S1.** Supplemental tables and figures for “HapAsmbl: A reference-aided pipeline for assembling haplotypes in Nanopore amplicon sequence data of polymorphic populations”

**Table S1**. Perennial ryegrass germplasm used in this study.

| ID | NZ cultivar | Country of origin of exotic parent (identifier) | Experiment used for (no. of plants) | |
| --- | --- | --- | --- | --- |
| One^50^ | One^50^ | NA | Protocol design (5)  Haplotype diversity (14) | |
| F2 A24983 | Stella | Algeria (P I231590) | Protocol design (5) | |
| F2 A24980 | Abermagic | Denmark (P I200322) | Haplotype diversity (21) | |
| F2 A24992 | Abermagic | Sweden (P I265335) | Haplotype diversity (12) | |
| F2 A24993 | Arrow | Sweden (P I265335) | Haplotype diversity (12) | |
| F2 A24994 | Abermagic | Sweden (PI303014) | Haplotype diversity (31) | |
| F2 A24995 | Stella | Sweden (P I303037) | Haplotype diversity (14) | |
| F2 A25007 | Abermagic | Lithuania (P I502414) | Haplotype diversity (19) | |
| F2 A25008 | Arrow | Lithuania (P I502414) | Haplotype diversity (20) | |
| F2 A25012 | Prospect | Sweden (P I303037) | Haplotype diversity (12) | |
| *Note:* NA = not applicable; NZ = New Zealand. | | | |  |

**Table S2**. Gene identifiers for genes targeted for amplicon sequencing. Identifiers are based on the Kyuss_2.0 reference genome (Chen et al., 2024).

| Gene name | Gene identifier |
| --- | --- |
| *FLOWERING LOCUS T 3* (*FT3*) | *LOC127313630* |
| *FLOWERING LOCUS T-LIKE 9* (*FTL9*) | *LOC127326949* |
| *VERNALIZATION 1* (*VRN1*) | *LOC127292808* |
| *VERNALIZATION 2A* (*VRN2A*) | *LOC127297393* |
| *VERNALIZATION 2B* (*VRN2B*) | *LOC127297489* |
| *CONSTANS* (*CO*) | *LOC127317264* |

**Figure S1**. Diagrammatic overview of regions in five perennial ryegrass flowering-time genes assayed using long-read amplicon sequencing. The figure shows a map of primers flanking regions of (A) CO, (B) FT3, (C) FTL9, (D) pVRN1 and piVRN1 (p: promoter; pi: promoter with regions of intron 1), (E) pVRN2A, and (F) VRN2B. The primer sequences and the experiments in which they were used are shown in Table S3. Negative and positive numbers indicate the location of a forward and reverse primer, respectively, relative to the start codon (0) in base pairs. Thick bars in each gene model represent exons. F: forward primer, R: reverse primer. Sequences and annotations are based on the Kyuss reference genome (Frei et al., 2021).

**Table S3**. Sequences of gene-specific primers targeting regions within six perennial ryegrass flowering-time genes used in protocol design and haplotype diversity studies.

| Target gene | Forward primer sequence | Reverse primer sequence |
| --- | --- | --- |
| **Protocol design** | | |
| *FT3 (F2)* | CGTTAAAGCCGTTCATGTGTAAC | GTTGTGGTGGTGTCAATTATAC |
| *FTL9* | ATGGATAGGGATGCAAGTGT | GTGATACAGCACTACCATACC |
| *pVRN1*  *(F1-R1)* | ATGACCGCATGTGACCTACA | GAACAACAAGGGTTCCGTTC |
| *pVRN2A* | GTGGAAAAGGCTCAGTGTGG | ACGGGGAACACGCTTATGTT |
| *VRN2B* | TCAGGATTGTGTGGGTCGAT | CTCATGAACACTATTCATTGGAGGA |
|  |  |  |
| **Haplotype diversity** | |  |
| *CO* | TCTGTCAACAACAGCATATCG | CATACCACACAATTATTCAGAGCAC |
| *FT3 (F1)* | CCAGAATCCATGTTGCAGCT | GTTGTGGTGGTGTCAATTATAC |
| *FT3 (F2)* | CGTTAAAGCCGTTCATGTGTAAC | GTTGTGGTGGTGTCAATTATAC |
| *piVRN1*  *(F1-R2)* | ATGACCGCATGTGACCTACA | CATGAGGTCGCTACACTGTTTGG |
| *piVRN1 (F2_R2)* | GGATACATCATTGGCTACCTTTTG | CATGAGGTCGCTACACTGTTTGG |
|  |  |  |

Note: p = promoter; pi = promoter-intron.

**Figure S2**. Quality control steps. (A) Workflow for post-sequencing base calling, read quality control, and filtering applied to Oxford Nanopore Technologies (ONT)-based long-read amplicon sequencing (LRAS) data prior to haplotype assembly. (B) Additional quality control steps incorporated into the HapAsmbl pipeline to remove concatenated amplicons.

**Table S4.** Comparison of tools for quality control and HapAsmbl for ONT R9.4.1 and 10.4.1 flow cell chemistries.

| Step | R9.4.1 ONT technology | R10.4.1 ONT technology |
| --- | --- | --- |
| **Quality control steps prior to haplotype assembly** | | |
| Post-sequencing calling, demultiplexing and adapter trimming | Latest ONT proprietary basecaller and trimmer | Latest ONT proprietary basecaller and trimmer |
| Split chimeric reads containing within-read adapters | Duplex tools v0.2.7 or higher | Duplex tools v0.2.7 or higher |
| Removal of residual terminal adapters (OPTIONAL) | Porechop v2.04  (legacy tool not maintained since 2018) | Porechop_ABI  (or alternative trimmer capable of auto-detecting ONT adapter sequences) |
| Removal of low-quality and reads shorter or longer than expected amplicons | Filtlong v0.2.1 or higher | Filtlong v0.2.1 or higher |
| **HapAsmbl** | | |
| Map FASTQ files to reference sequence | Minimap2 v2.23 or higher | Minimap2 v2.23 or higher |
| Additional quality control |  |  |
| - removal of unmapped, non-primary and secondary alignments | SAMtools v1.15 or higher | SAMtools v1.15 or higher |
| - removal of fused or ligated amplicons | BBMap v39.01 or higher | BBMap v39.01 or higher |
| Call small variants (SNPs and small indels) | Clair3 v1.05 or higher  (with model r941_prom_sup_g5014) | Clair3 v1.0.5 or higher  (with model r1041_e82_400bps_sup_v500) |
| Phase into haplotypes | WhatsHap v1.2 or higher | WhatsHap v1.2 or higher |
| Clustering | WhatsHap v1.2 or higher | WhatsHap v1.2 or higher |
| Draft consensus sequences | SPOA v4.1.0 or higher | SPOA v4.1.0 or higher |
| Polish draft consensus | Flye v2.9 or higher  (with flag --nano-raw) | Flye v2.9 or higher  (with flag --nano-hq) |

**Figure S3**. Gel electrophoresis of the PCR products of five ryegrass flowering-time genes (FT3, FTL9, pVRN1, pVRN2A, VRN2B) from gDNA from five plants from the F_2_ A24983 population (plants F1, F9, F10, F12, F18, and a pooled sample) and One^50^ (plants O1, O2, O3, O5, O11, and a pooled sample). Primer locations are shown in Figure S1 with expected amplicon sizes of 1784 bp for FT3, 2825 bp for FTL9, 2291 bp for pVRN1, 2482 bp for pVRN2A, and 4155 bp for VRN2B based on the Kyuss reference genome (Frei et al., 2021). For each sample, two PCR reactions were performed for each primer pair to increase the chances of obtaining a high concentration of purified products after gel extraction. For FT3 and FTL9, PCR reactions were loaded as single replicates consecutively on the gel, while for other genes, PCRs of a given sample were loaded sequentially as duplicates. Marker sizes are indicated.


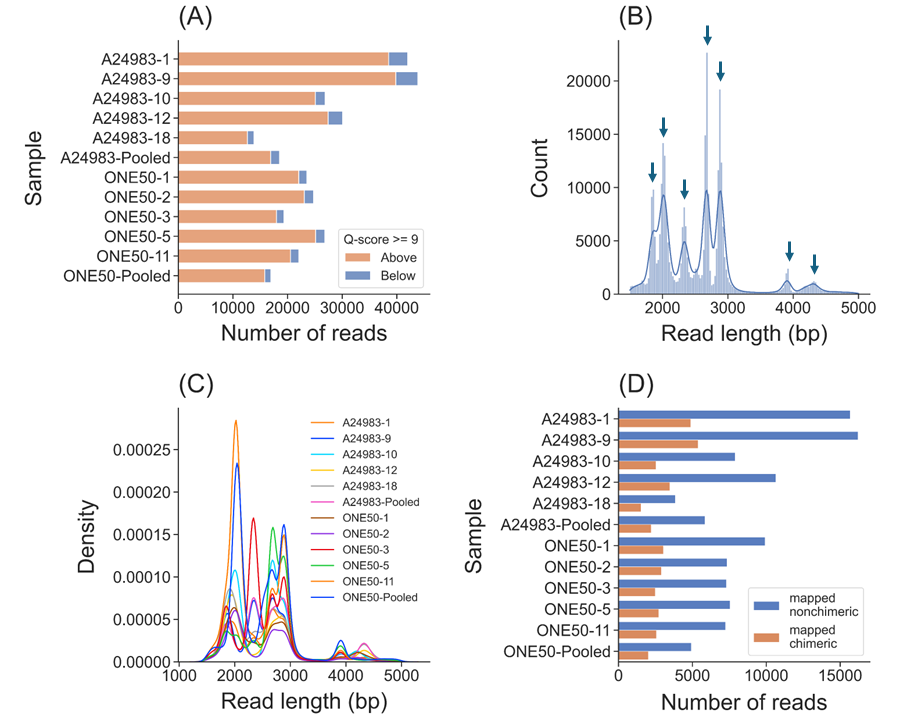


**Figure S4**. Key results of amplicon sequencing of ryegrass flowering genes using ONT sequencing. (A) Total number of raw unfiltered reads per sample below and above a minimum Q-score of 9. (B) Histogram of reads between 1500 and 4500 bp in the raw multi-locus amplicon dataset. Arrows above each peak indicate read lengths matching the approximate sizes of the target amplicon. From left to right: 1784 bp for FT3, 2291 bp for pVRN1, 2482 bp for pVRN2A (lower allele), 2825 bp for FTL9, 4000 bp for pVRN2A (upper allele), and 4155 bp for VRN2B. (C) Distribution of reads between 1500 and 4500 bp per sample prior to pre-assembly quality control. Double peaks are likely due to size variation between alleles resulting from large indels. (D) Number of non-chimeric and chimeric reads per sample that mapped to a reference gene after the pre-assembly quality control.


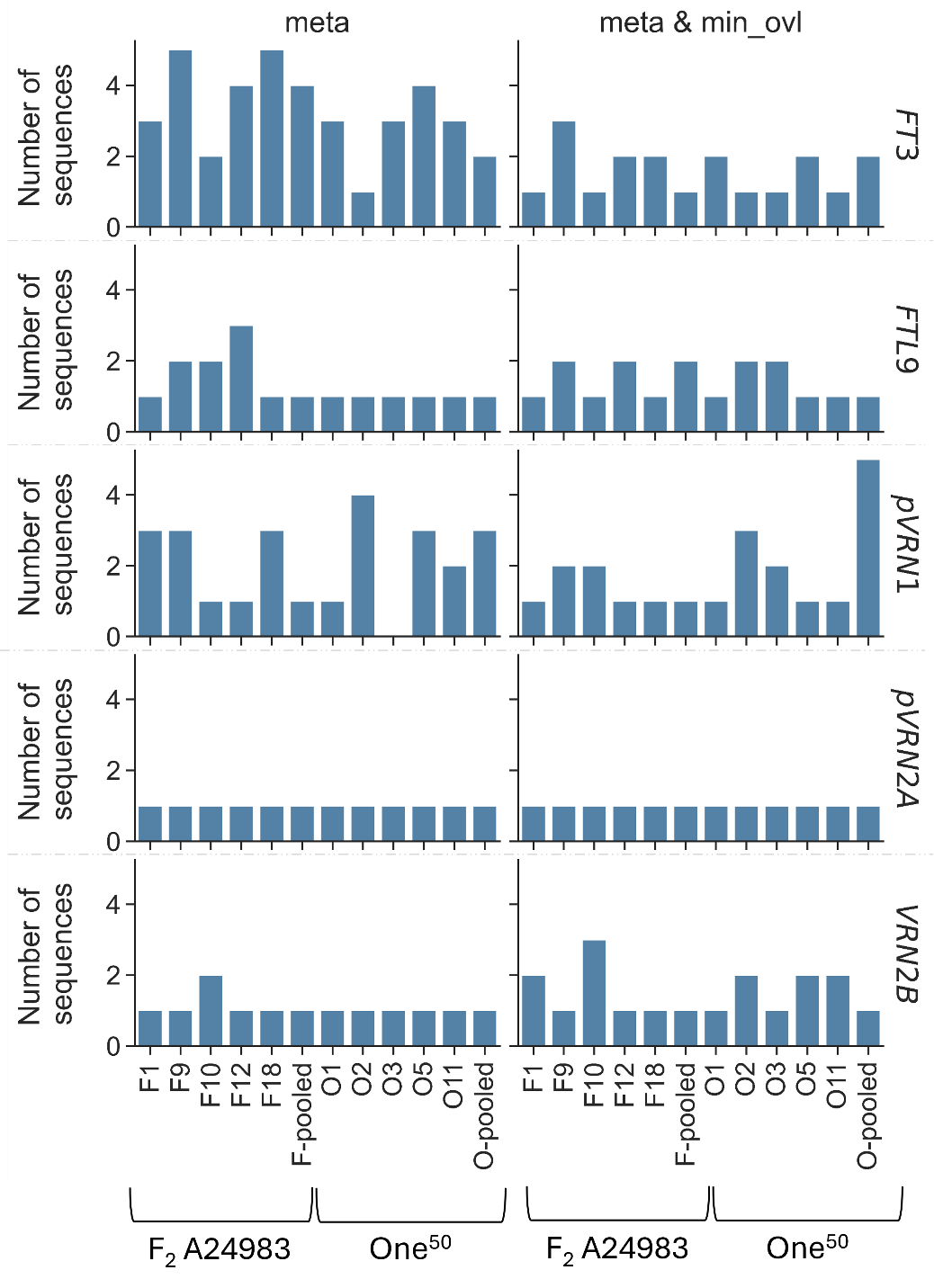


**Figure S5**. Results of haplotype assembly using the de novo method. The figure shows the total number of sequences assembled from amplicon sequences of each target gene (right) for each sample using Flye’s de novo assembly algorithm. The column name (top) indicates the run parameter: meta specifies that data has uneven coverage, while min_ovl indicates a minimum read overlap of 1000 bp among reads. Six gDNA samples (five individual plants and one pooled) were each prepared for two perennial ryegrass populations, F_2_ A249843 and One^50^. Plants used in preparing individual samples were diploid (maximum of two alleles). The pooled gDNA sample was prepared from pooled leaf materials of the five individual plants. Sequences are provided in Appendix S2.


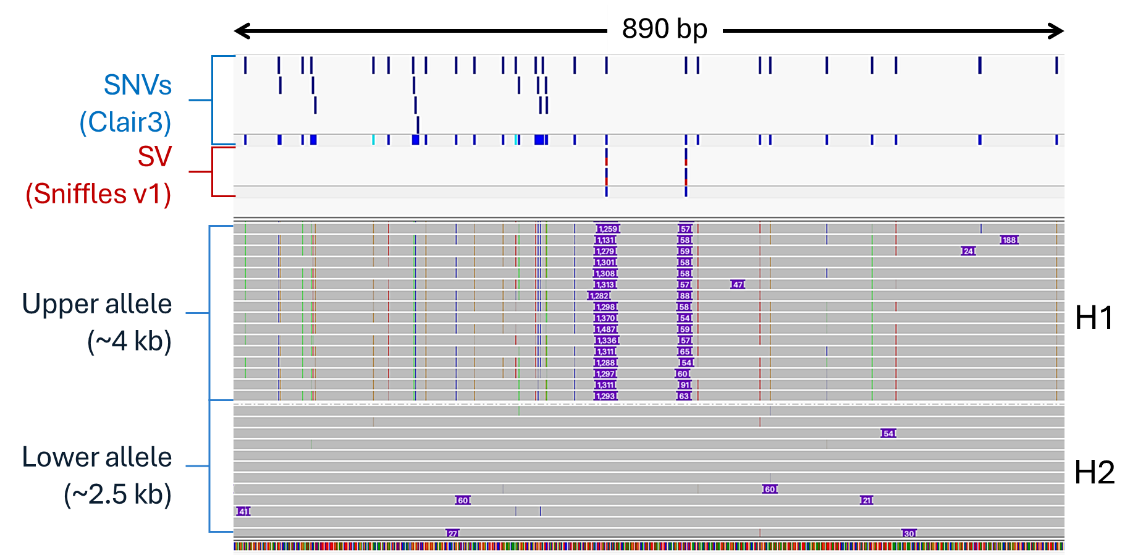


**Figure S6**. Genetic and haplotype variation within a ~900-bp region of the VRN2A promoter of the F10 plant. The pVRN2A reads cluster into two predominant haplotypes, designated as H1 and H2. The black box highlights a ~170-bp region where the two haplotypes can be differentiated by two large indels (a ~1300-bp region and a ~50-bp region) and numerous SNPs as shown in Table 1.

**Figure S7**. Results of the reference-aligned haplotype assembly using a Clair3-WhatsHap-BCFtools workflow. Row labels represent sample names while column labels represent target genes. Assemblies of each sample have been mapped to the allele of the corresponding gene from the Kyuss reference genome (Frei et al., 2021). Gray bars represent sequences. Colored lines represent variant bases relative to the allele in the Kyuss reference genome. Thick purple bars represent structural variants. The identity of genes (pVRN1 and VRN2B) with a high number of differences to the reference genes was confirmed by BLAST. Haplotype sequences are provided in Appendix S3.

**Figure S8**. Impact of filtering of ligated amplicons with BBMap prior to haplotype assembly. The image shows pairwise alignments of ONT amplicon reads of pVRN1 from the F1 plant from the F_2_ A24983, and the allele from the Kyuss reference genome (Frei et al., 2021). (A) Alignment shows ligated amplicons in LRAS data produced during library preparation for ONT sequencing prior to filtering with BBMap. (B) Pairwise alignment of reads from the same sample after removal of ligated amplicons with BBMap as an additional quality control step incorporated into the HapAsmbl pipeline. Multi-colored portions at the end of each bar represent “foreign” sequences flanking the target sequence on either the 5′ end, 3′ end, or both ends of the read. Gray bars that are not flanked by multi-colored segments represent non-chimeric reads.


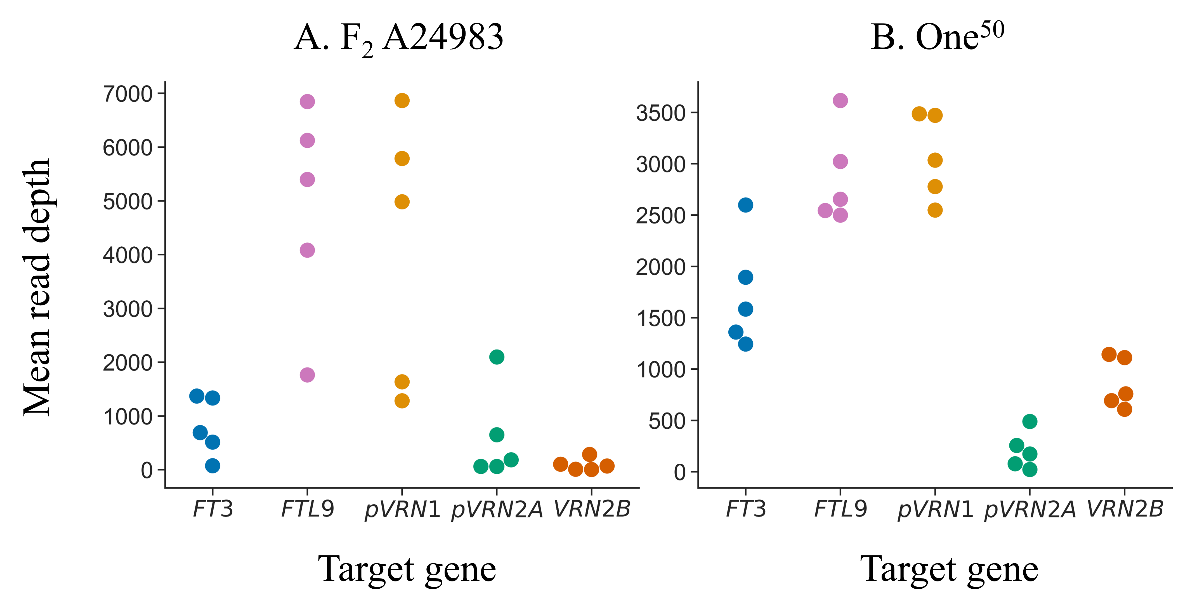


**Figure S9**. The mean depth of sequencing of each target amplicon for samples in F_2_ A24983 (A) and One^50^ (B) ryegrass populations after removing chimeric sequences in the HapAsmbl pipeline. Each dot represents a gDNA sample, and different colors represent amplicons produced from different genes.

**Figure S10.** Results from the HapAsmbl pipeline. Row labels represent sample names, while column labels represent target genes. Assemblies of each sample have been mapped to the allele of the corresponding gene from the Kyuss reference genome (Frei et al., 2021). Gray bars represent sequences, colored lines represent variant bases relative to the allele in the Kyuss reference genome, and thick purple bars represent structural variants. The identity of genes (pVRN1 and VRN2B) with a high number of differences to the reference genes was confirmed by BLAST. Haplotype sequences are provided in Appendix S4.

**
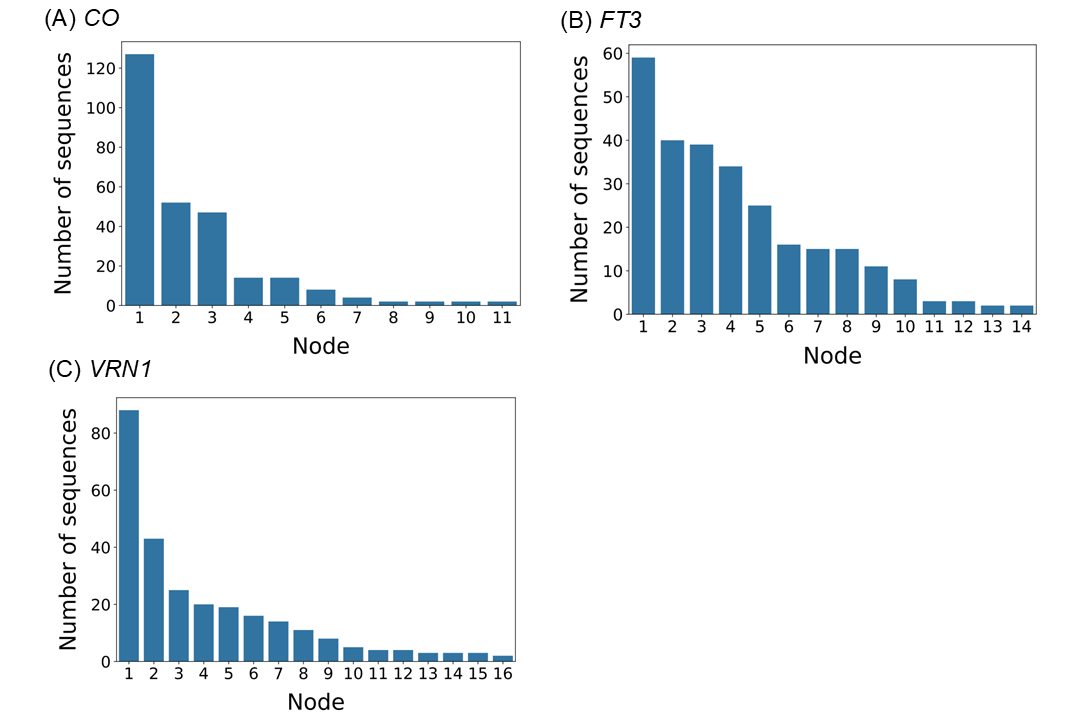
**

**Figure S11.** Distribution of unique sequences (nodes) across three floral genes—*CO* (A), *FT3* (B), and *VRN1* (C)—in 141 F_2_ perennial ryegrass plants. Plants were sampled from eight diverse F_2_ ryegrass populations and assayed using a multi-locus LRAS-HapAsmbl workflow developed in this study. Unique alleles per gene and allele frequencies were analyzed using PopART v1.7 (Leigh and Bryant, 2015). Nodes are sorted based on size in descending order.

**REFERENCES**

Chen, Y., R. Kölliker, M. Mascher, D. Copetti, A. Himmelbach, N. Stein, and B. Studer. 2024. An improved chromosome-level genome assembly of perennial ryegrass (*Lolium perenne* L.). *GigaByte* 2024: e112.

Frei, D., E. Veekman, D. Grogg, I. Stoffel-Studer, A. Morishima, R. Shimizu-Inatsugi, S. Yates, et al. 2021. Ultralong Oxford Nanopore reads enable the development of a reference-grade perennial ryegrass genome assembly. *Genome Biology and Evolution* 13: evab159.

Leigh, J. W., and D. Bryant. 2015. PopART: Full-feature software for haplotype network construction. *Methods in Ecology and Evolution* 6: 1110–1116.
